# Concussion competencies: a training model for school-based concussion management

**DOI:** 10.2217/cnc-2018-0008

**Published:** 2019-02-11

**Authors:** Arthur Maerlender, Jonathan D Lichtenstein, Jennifer Parent-Nichols, Kate Higgins, Peggy Reisher

**Affiliations:** 1 University of Nebraska, Center for Brain, Biology & Behavior C 86 East Stadium, Lincoln, NE 68588-0156, USA; 2 Director, Pediatric Neuropsychological Services Dartmouth-Hitchcock Medical Center, Assistant Professor of Psychiatry, Geisel School of Medicine at Dartmouth HB 7750 Department of Psychiatry One Medical Center Drive Lebanon, NH 03756, USA; 3 Assistant Professor Franklin Pierce University 670 North Commercial St. Manchester, NH 03101, USA; 4 Brain Injury Alliance of Nebraska PO Box 22147 Lincoln, NE 68542, USA

**Keywords:** concussion competencies, concussion education, school-based concussion management

## Abstract

This study reports on the use of ten knowledge competencies related to the behavioral management of concussion in schools. Trainings using these competencies as learning objectives were delivered to school personnel. This aims of the use of competencies in this way are to streamline the education of key stakeholders, to establish clear roles and responsibilities for constituents and equip individuals working with students following a concussion with the relevant knowledge to optimize outcomes. The majority of participants, primarily speech language pathologists working as related service providers in the schools where the trainings occurred, judged the use of the competencies to be informative and useful to their practice both immediately following the training and at a 5-month follow-up. The greatest gains in knowledge were noted by those participants self-reporting the least amount of knowledge pre-training. Participants also ranked the perceived value and relative importance of each of the ten competencies.

## Background

Pediatric and adolescent concussions commonly are managed in school settings, often by non-medical professionals. The school environment can pose several challenges to the student recovering from concussion: bright lights, loud noises and the demand for cognitive and physical effort may result in symptom exacerbation. Academic and environmental adjustments may be necessary during the recovery period to encourage safe behavior of the child or adolescent in returning to their role as student. To be effective in these efforts, school personnel need appropriate information and specialized training for addressing these challenges. Accordingly, experts are calling for increased resources to be directed for proper concussion education within the school environment [1].

Evidence supporting ‘active rehabilitation’ strategies, including a graduated return to cognitive and physical exertion, necessitates the involvement of school personnel [2]. For this reason, school-based concussion management programs with a focus on supporting students as they ‘return to learn’ are becoming more common. In fact, a recent White Paper published in the Journal of Head Trauma Rehabilitation reports that *“states cannot afford not to implement a reasonable supports program, as the lack of adequate academic supports had led to litigation in several states”* [3]. Adequate training of school staff is necessary to ensure competence in the delivery of these services.

In the early stages of a brain injury, proper medical identification and intervention are critical. However, once a brain bleed or lesion has been ruled out and there is no imminent danger, concussion management is primarily concerned with secondary prevention. In all settings, secondary prevention for concussion is accomplished through careful monitoring and management of behavior and status through gradual, tolerated increases in cognitive and physical activity [1]. The steps taken to avoid re-injury, prevent worsening of initial injury, and ensure that recovery is not prolonged are primarily behavioral in nature.

Educational staff is already familiar with addressing student needs from a behavioral perspective. Such familiarity may come from general classroom experience and work within specific support models such as response to intervention and positive behavioral support (PBS) [4–6]. Response to intervention is an educational approach designed to identify and provide support to students with learning and behavioral needs. PBS has become the most utilized and effective method of behavioral management in schools. The roots of PBS lay in behavior analysis [7]. Thus, school personnel may already be equipped with techniques to modify behavior, make academic and environmental adjustments to target areas of challenge and monitor response to applied interventions. Still, many school personnel likely lack specific knowledge regarding brain injury that is required to apply pre-existing skill sets to the particular needs of the recovering student in the academic setting [8].

Education is necessary to support schools in their efforts to transition students recovering from concussion safely and efficiently back to the academic environment. Schools require direction regarding such practice. School personnel must have training regarding their district's plan for managing the students returning to the classroom [9].

While all 50 states have laws that require training for some personnel managing students with concussion and for the students themselves, the scope of this knowledge and training has not been defined. Several programs have been developed in the USA to respond to the needs of students recovering from concussion identified by the American Academy of Pediatrics (AAP) [10]. Although highly commendable and promising, the effectiveness of these programs is yet to be systematically examined.

Recently, the AAP formed a consensus panel to identify areas that required immediate attention in pediatric concussion, particularly in regards to institutional inititatives [11]. The results of the process identified the critical elements of concussion programs, including, the neurophysiology of mTBI, the effects of mTBI in school, recovery from mTBI, program or institutional policy considerations, the formation of interdisciplinary teams and the different ‘constituency’ roles, injury identification, assessment and progress monitoring protocols, the evaluation of mTBI symptom status, academic, physical and emotional interventions and ‘accommodations’ and coordination of medical-to-school communication. The panel encouraged enhanced professional development and training around these elements [11].

Independently and with similar timing, the authors of this project developed a model for training school personnel through a grant-funded project through the Nebraska Department of Health and Human Services [12]. To provide a guide for information delivery, ten competencies were developed to provide advanced training for school personnel already managing concussed students. Those competencies address the essential topics identified in the AAP paper [11]. More recently, the authors have published an expanded manual reflecting these competencies [13,14].  While there was no direct coordination between the two groups, the overlap of topics suggests convergent ecological validity for these ideas.

One aim in using these ten competencies in delivering trainings was to begin to define the relative importance of knowledge in different domains or competencies of concussion management. This process could help to inform and streamline the education of key stakeholders (constituent) in this area. While it has been recommended that all personnel receive training around their district's management plan [9], not all school personnel involved in concussion management likely require the same set of skills. The development of appropriate competencies specific to key stakeholders in concussion management may provide a common language, create a focus for delivery of critical content, assist with assessment of learning and promote positive changes in educational practice. Furthermore, the use of a set of standardized competencies may provide an opportunity to assess systematically the impact of such trainings on students recovering from concussion.

Core competencies have been defined as the essential minimal set of a combination of attributes, such as applied knowledge, skills and attitudes, which enable an individual to perform a set of tasks to an appropriate standard efficiently and effectively [15]. The delineation of these competencies informs education, practice and research. Competencies provide a common, shared language for defining what one is expected to know and do in order obtain optimal clinical results and should be a priority in healthcare education [15,16]. A recent consensus statement regarding core competencies in evidence-based practice for health professions stated, *“A clear outline of core competencies is critical in any healthcare education setting, as it informs the blueprinting of a curriculum, including learning outcomes, assessment strategies and graduate attributes”* [17].

This paper reports on steps in the initial development of trainings around a suggested set of ten competencies. These competencies aim to establish requisite knowledge, skills and abilities for school personnel involved in coaching students toward successful reintegration to the academic environment following concussion. Ultimately, they are designed to establish clear roles and responsibilities for key stakeholders and equip individuals working with students following a concussion with the relevant tools and knowledge necessary to optimize outcomes. These ten suggested competencies for the described trainings were developed by three experts in the field with over 100 years of combined medical and classroom experience. These suggested competencies are in agreement with the guidelines for diagnosis, prognosis and management treatment of pediatric mTBI set by the Centers for Disease Control and Prevention (CDC) in 2018 with respect to school personnel [18]. The competencies address biological aspects of concussion, behavioral factors and programmatic considerations.

As experts in the field have identified the need to understand both the training needs of school staff working with students after concussion and the most effective formats of this training [2], this study describes the use of these ten core competencies with school personnel through education, testing and surveys. See Table 1 for a list of the Competencies; a more detailed description of the competencies can be found in the Appendix.

**Table 1.** **Concussion competency headings.**

**I. Biological aspects**

1. Basic neuroanatomy

2. Biomechanics of injury

3. Concussion basics

**II. Behavioral factors**

4. Risk factors

5. Prevention

6. Evaluation

7. Evaluation and assessment practices

8. Postacute and chronic treatment approaches

**III. Programmatic considerations**

9. Individual recovery and the role of the school

10. Programmatic concussion management

The aim of the current analyses were to confirm the usefulness of the specific competencies and to demonstrate an approach to using competencies for teaching and assessment. Data gathered from these trainings were used to assess gains in knowledge, gains in competency specific knowledge, and knowledge gains with respect to self-reported baseline knowledge of concussion prior to the trainings.

## Methods

### Participants

Institutional Review Board approval for the project was obtained. Four schools were invited and agreed to participate in the project: an urban public high school (grades 9–12, enrollment 1620), an urban public middle school (grades 6–8, enrollment 848), an urban parochial high school (grades 9–12, enrollment 1280) and a rural school (grades kindergarten to 12, enrollment 527). All schools were developing or had already developed concussion management teams and this training served to provide formal education to those teams. The urban school district requested that their Speech–Language Pathologists (SLP) be able to participate in this training. SLPs in this district were all highly involved in their schools’ concussion management programs. 102 participants received the offered training, out of which 96 completed both pre- and post-tests of knowledge. More participants completed a post-training survey (n = 101), than the follow-up survey (n = 71). Schools received a stipend for participation and completion of assessments. Results from participants completing the full training and both pre- and post-tests are reported here.

The final sample of 96 participants was primarily female (n = 82; 85%). By profession, the majority of the participants were SLP's (n = 70; 73%) and classroom teachers (n = 16; 17%). The remaining 10% of participants was comprised of school staff/administrators (n = 8; 8%) and school medical personnel (n = 2; 2%). Participants’ mean age was 39.44 (standard deviation [SD] = 12.6), with an average of 13.6 (SD = 11.7) years in their respective field. Most participants had earned a Masters’ degree (n = 76; 79%). All participants were asked to indicate their perceived level of knowledge of concussions before the training started, with 81 respondents. The sample was quite knowledgeable about concussions with 63 of 81 self-describing themselves as having an average or above average knowledge level (78%). See Table 2 for additional demographic information.

**Table 2.** **Demographics of sample (n = 96).**

		**n**	**%**
Highest degree completed			

	Bachelors	17	18

	Masters	76	79

	Other	3	3

	Total	96	100

Position		n	%

	Teacher	16	17

	SLP	70	73

	School medical personnel	2	2

	School staff/administrator	8	8

	Total	96	100

Perceived knowledge		n	%

	Below average	18	19

	Average	41	43

	Above average	22	23

	Missing	15	15

	Total	96	100

Years in field		n	%

	0-2 years	22	23

	3–7 years	15	16

	8–15 years	21	22

	15+ years	36	37

	Missing	2	2

	Total	96	100

### Instruments

#### Surveys

Prior to the training, participants completed a self-rating of perceived concussion knowledge (3 = below average prior knowledge of concussions; 2 = average amount of knowledge; and 3 = above average amount of knowledge).

Immediately following the training, participants were asked to respond to a satisfaction survey regarding the training. Additionally, they were asked to rank the importance of the ten competencies in relation to their work experiences. Specifically, participants were asked ‘In the list below, indicate the usefulness of the material under each of the ten core competencies’ as useful and informative, neutral or not useful.

Finally, a follow-up survey with slightly different response choices was administered at 5 months. In this ‘competency relevance ranking survey’, participants were asked to rank order the competencies in terms of their perceived usefulness over the past 5 months. Finally, a three-item survey with four responses regarding degree of satisfaction with the training were administered at the end of training.

#### Knowledge assessment

An assessment of knowledge related to the competencies was created and administered before and after training. Two 40-item test forms were developed from a pool of 75 items related to the ten competencies. A team of experts in education and concussion developed the items. Some items appeared on both forms to ensure the ten developed competencies were represented equally by questions across both forms. Division of the test items served to permit adequate time for the trainings, decrease the length of the assessment for any individual and allow for a more robust investigation of learning.

### Procedure

Prior to the training at any school, participants completed an initial survey of perceived concussion knowledge and an assessment of knowledge related to the ten suggested competencies. Participants were assigned randomly to complete either version A or B of the assessment. The training following this assessment covered material related to the ten proposed competency areas. The guidelines for diagnosis, prognosis and management treatment of pediatric mTBI set by the CDC in 2018, as appropriate for school staff, further informed the trainings [18]. Presentation of information occurred in a number of formats to facilitate learning. PowerPoint slides, vignettes representing actual classroom situations to provide practice, question and answer sessions and activities to promote self-reflection were offered. Attention was given to the provision of opportunities for active learning throughout the trainings. To facilitate individual school scheduling needs, the trainings were completed in two, 2–3 h sessions totaling between 4 and 5 h.

Immediately following the training, participants completed the same version of the assessment they had completed prior to the training. 48 participants completed both pre- and post-tests for form A and 48 completed both pre- and post-tests for form B. Based on school preferences and logistical concerns, some participants took the pre-test only via online survey system (Qualtrics). Links were mailed directly to participants several days before the training (3–4 days). All post-tests were taken at the final presentation site.

Two questions on version B were found to have typographic errors (after administration) that rendered the response options invalid. Those questions were removed, leaving 38 questions in version B and 40 questions in version A. In analysis, the percent correct was calculated for each form. Because of school constraints, the interval between pre- and post-assessment administrations ranged from 2 weeks to 1 month, with an average of approximately 2 weeks. That is, pre-tests were taken at differing times within a 1-week window, and some post-tests were taken after the training via email.

Immediately following the last training session, participants were asked to respond to a satisfaction survey regarding the training. Additionally, participants were asked to rate the ten competencies based on their perceptions of usefulness in coaching students back to learning after concussion. A second survey of competency relevance was sent to all participants 5 months following the completion of the training via email. The follow-up survey was administered 6 weeks into the following school year. The follow-up survey prompt was ‘This training was organized around ten core competencies. In the list below, indicate the usefulness of the material under each of the ten core competencies’.

### Data analysis

The competency surveys were tabulated to reflect the participants’ perception of the effectiveness of the competency structure for application to their own real-life work in this domain. The survey immediately following the training categorized the perceived usefulness of the material under each of the ten core competencies. The percent of participants who rated each competency as useful was calculated. In the 5-month follow-up competency relevance ranking survey, the percent of participants who ranked each competency as one of the top three (greatest usefulness) was tabulated.

For the pre- and post-knowledge assessment, the percent correct for each version was calculated and used as means to understand learning relative to the trainings. Percent correct scores for test items related to each competency were also calculated by summing the competency item scores and dividing by the number of items that had assessed that competency. Demographic factors were assessed with a series of ANOVAs and regression models to evaluate systematic differences in the assessment scores: current position, years in the field, highest degree obtained, age, sex and site of training. Age and years in the field were independent variables in a regression model, while sex, current position and highest degree obtained were analyzed in separate ANOVAs. A repeated measures analysis of variance with test version as the between subject's factor (to control for slight differences in test versions), and pre-test to post-test percent change as the within subjects’ factor, was the primary analysis.

As an indicator of validity, it was hypothesized that those participants reporting ‘below average’ knowledge regarding concussion would score lower on the pre-test than those participants reporting ‘average’ or ‘above average’ knowledge. Additionally, those participants with below average knowledge would have larger percent score increases from pre- to post-test. This hypothesis was assessed using an ANOVA. Scores for each of the ten competencies were calculated as the average of scores for the sum of competency items in the test. A within subjects t-test from pre- to post-test was calculated for each competency. Alpha was set *a priori* at p < 0.05. IBM's Statistical Package for Social Sciences version 23 was used and for all analyses. Finally, overall satisfaction of training surveys was tabulated as the percent agreement with four statements about the training and content.

## Results

### Surveys

To assess the impact of the use of the ten core competencies, results of the post-training survey and the 5-month follow-up survey were tabulated. With regard to the post-training survey, 79% of respondents indicated that the trainings were ‘useful and informative’ across the suggested competencies. No greater than 6% of participants judged any of the proposed competencies as ‘not useful’. Greater than 90% of participants rated the competencies of individual recovery, treatment approaches and  risk factors as useful to their practice. Risk factors and individual recovery were the highest rated competencies. Results of this survey are presented in Table 3.

**Table 3.** **Survey results of competency relevance.**

**Module**	**Post-training percentage rating of useful and informative (n = 101)^†^**
(1) Basic neuroanatomy	0.680

(2) Biomechanics of Injury	0.750

(3) Concussion basics	0.891

(4) Risk factors	0.901

(5) Prevention	0.860

(6) Evaluation	0.891

(7) Assessment practices	0.852

(8) Treatment approaches	0.929

(9) Individual recovery	0.940

(10) Programmatic concussion management	0.881

^†^Indicate the usefulness of the material under each of the 10 core competencies. Useful, neutral, not useful.

The 5-month follow-up survey was structured differently from the post-training survey due to its retrospective nature. The competency relevance ranking survey asked participants to rank order how useful each competency had been in their practice since the training. 71 (73.96%) participants responded to this follow-up survey. The percent of times the competency was ranked in the top three places were calculated. In the 5-month follow-up survey, the competencies of concussion basics, Treatment Approaches and Individual Recovery were ranked most consistently in the top three rankings.

### Assessment of knowledge

From the sample of 96 participants who completed the training and both pre- and post-tests, 48 (50%) participants completed version A and 48 (50%) different participants completed version B. None of the demographic variables contributed unique variance to the outcomes. A repeated measures, within-subjects ANOVA revealed a significant pre- to post-test difference in percent score change with a medium effect size: F(1.94) = 67.48; p < 0.001; η^2^ = .418. However, the form by change score was not significant: F(1.94) = 0.465; p = 0.497; η^2^ = .005. Overall, the mean percent change from pre- to post-test scores was 5.65%. Means, SD, minimum scores and maximum scores of pre- and post-test percent correct (combined form) are presented in Table 4. Distributions of both sets of scores had acceptable skew and kurtosis at pre-test, with a post-test shift to the right as expected.

**Table 4.** **Characteristics of pre-test and post-test scores (n = 96).**

**Statistic**	**Pre-test correct percentage**	**Post-test correct percentage**
Mean	0.797	0.860

Std deviation	0.086	0.082

Skew	-0.843	-1.308

Kurtosis	1.941	3.076

Minimum	0.450	0.530

Maximum	0.950	1.000

84% of participants had complete test results and provided responses to the self-rating of perceived concussion knowledge (81/96). Overall, pre- to post-test score change showed significant improvement: F(1.78) = 40.67; p < 0.001. Within-subject changes by group were not significant: F(2.78) = 1.227; p = 0. 299. However, post-hoc comparison of the mean of the change scores (Bonferonni corrected) was significantly different by group: F(1.2) = 9.923; p = < 0.001. The mean of the change score for the below average group was significantly greater than the change score for both the average group (mean change = 0.073; p = 0.002) and the above average group (mean change = 0.093; p < 0.001).

As expected, the below average group changed more from pre- to post-test than the other two groups. The absolute percent change within each group was as follows: above average = 4% increase, average = 6% increase and below average = 7% increase. The effect sizes for mean amount of change between groups was as follows: above average compared to average mean change difference = 0.02, d = 0.266; above average to below average mean change difference = 0.09, d = 0.967; average to below average mean change difference = 0.07, d = 0.792.

Competency test-item change scores (items related to specific competencies) were significant in four competencies. Biomechanics of Injury (p < 0.001), evaluation (p < 0.001), treatment approaches (p < 0.001) and individual recovery (p < 0.001).

### Satisfaction with training

Perceived value of the trainings for the whole group was uniformly high. 93% reported the training gave them a better understanding of the ‘how and why’ of concussion management. 86% rated they would utilize the information from the presentations when working with students with concussion. 88% were satisfied with the trainings in general.

## Discussion

All 50 US states have laws regarding concussions in youth sports that require education and training in concussion management for sports-related personnel. Only nine states have legislation requiring schools to have a protocol for managing concussed students as they return to academics. Education is a first step in providing school personnel with the knowledge necessary to the proper management of students returning to the classroom after concussion. The CDC recommends educating stakeholders [18,19]. However, there are no established curricula for directing the training of school personnel who work with students with concussions. The 4–5h, competency-based, concussion management training described in this paper was developed to leverage existing educational, behavior-based approaches to make school-based concussion management accessible.

Despite the high level of baseline knowledge of this group, the competency-based training was judged as useful and informative to practice with students by the majority of participants, comprised primarily of speech language pathologists. The fact that related service providers working in schools possessed a high level of pre-training knowledge and found the competency-based trainings useful was encouraging.

Following the training, the competencies of risk factors, individual recovery and treatment approaches were identified as most useful by the participants. 5 months following the training and after participants had the opportunity to apply their learning to their practice, the competencies of concussion basics, individual recovery and treatment approaches were reported to be the most useful in the context of school-based concussion management. The competencies of basic neuroanatomy and biomechanics of injury were judged the least useful.

Knowledge improvement was statistically significant with a medium effect and significantly larger effect for those with the least pre-training knowledge. Comparison of knowledge change by self-perceived knowledge provided an opportunity to assess the incremental validity of the knowledge assessment. While these knowledge ratings were self-reported, significantly greater learning was documented for those with less pre-existing knowledge.

When assessing learning across each competency from pre- to post-test, the greatest learning was found to be in the competencies of biomechanics of injury, evaluation, treatment approaches and individual recovery. This finding is comparable to those competencies most participants identified as helpful to their practice, both immediately following and 5 months following the training. Specific discrepancies between participant perception of importance of the suggested competencies of basic neuroanatomy and biomechanics of injury and actual knowledge gained could have several explanations. Literature suggests that educators lack training regarding traumatic brain injury (TBI) and therefore may be unaware of the mechanisms, related recovery, and supports that may be necessary for students returning to the classroom after concussion [8]. If educational personnel have not been exposed to such information, growth in learning may be anticipated in those areas after training. Additionally, as adult learners, school personnel seek knowledge that is immediately applicable to their school settings, and they are drawn to material they perceive as relevant to their concept of self and as promoting job performance [20–22]. Supporting this explanation, treatment approaches generated the most change in learning and was seen as useful.

This paper describes a first step in determining competencies necessary for school personnel to coach students in safe and efficient return to the educational environment. The continued investigation and development of these suggested competencies is warranted in light of these findings. The development of a defined requisite knowledge, skills and abilities in educational, behavior-based concussion management may allow for a more fine-grained analysis of teaching and assessment of learning for school personnel that has not been present in concussion management. Furthermore, a use of competencies to define a specific scope of knowledge for very busy school personnel may provide a way to identify streamline necessary knowledge by role.

## Limitations

This study was not without its limitations. The sample of participants in this initial investigation was one of convenience. As such, the population was highly representative of speech language pathologists. This sample limits generalizability of our training to teachers, parents and other related service providers and allied health professionals. While the study sample did include classroom teachers, school administrators and school medical personnel, they represented the minority of participants. As such, the findings of this study cannot necessarily be generalized to educators more broadly. While the role of speech language pathologists as team leaders in concussion management may not describe typical practice across all school districts, nor is it endorsed by the authors as best practice, it represents actual practice within these districts. Related service providers, such as speech language therapists, occupational therapists and physical therapists, are identified as critical members of the academic team, responsible for providing support to students following concussion [2]. In this role, SLPs are trained to view student needs through an educationally relevant lens. Use of SLPs as participants in this investigation serves as a place to begin to understand the impact of the use of competencies to define roles and necessary knowledge.

Additional limitations include challenges with methodological consistency that did not always allow for regimented data collection practices. For example, some pre-tests were completed via paper and pencil, while others were completed via online survey. Although the change scores for each form was similar, the lack of form equivalence raises questions about the test itself. Furthermore, there was no control group in this study and the participants were not randomized. These elements were extremely challenging to execute within this sample and the study parameters. Lastly, while a 74% response rate is relatively high, there is the potential for response bias in the 5-month follow-up survey. It is possible that participants who did respond to this survey were highly motivated and potentially had greater investment in the learning and application of concussion management strategies presented in the trainings. Interpretation of findings related to this survey should be viewed in light of this potential bias.

This training and assessment were initial steps toward the development of concussion knowledge competencies. Further steps are needed, including a broad survey of constituencies involved in management of students returning to the classroom following concussion, comprehensive validation of the competencies by a larger panel of experts in the field and the establishment of behavioral anchors for tasks that are constituent specific. The validity of the use of concussion competencies may best be supported by direct assessment of job performance and functional outcomes following trainings.

## Conclusion & future perspective

The purpose of this project was to examine an initial set of core knowledge competencies that would facilitate a more detailed, systematic training in behaviorally based concussion management for school personnel. All of the ten competencies were rated as useful by training participants, although, as might be expected, the biological aspects of concussion were seen as less useful to the participants. Still, it is important to have an awareness of the biological realities of concussion as they help to develop an understanding of symptom experience and appropriate expectations for recovery. Gains in knowledge were consistent with ratings of usefulness.

Furthermore, those participants who reported below average knowledge prior to the training gained the most knowledge overall. Thus, the competencies provided a means for understanding specific gaps in stakeholder knowledge and proved to be a useful organizational tool. Certainly, additional competencies may be identified. However, the use of such competency categories is felt to improve the validity of trainings, and provide a consistent platform for research and evaluation.

The focus on behavioral aspects of managing concussions is a unique approach to understand concussion management practice and consistent with general school behavioral interventions. As there are no validated medical strategies for the management of concussion in the noncritical acute stage, behavioral management remains an important option. The behavioral model of concussion management requires further study, with the next step likely being a randomized controlled trial comparing the effectiveness of this approach to other curricula.

The intention of this project was to make an initial step in setting a benchmark of relevant categories of knowledge that is applicable within a behaviorally oriented setting (i.e., school). Furthermore, assessment of that knowledge based on relevance to practice helps establish criterion-related and ecological validity to the process. By defining an initial set of knowledge competencies, it becomes possible to better target specific constituencies with subsets of the broader corpus. For instance, school superintendents may not need to know about specific classroom adjustments, but likely need to understand concussion laws, as well as the procedures and best practice within their schools. School-board members, principals, athletic directors, classroom teachers, parents and students themselves have differing knowledge needs. The use of competencies, as suggested here, can assist in developing specific materials for each constituency group and creating the most efficient transfer of necessary knowledge for constituent practice.
